# Louisville Medical News

**Published:** 1878-02

**Authors:** 


THE "LOUISVILLE MEDICAL NEWS."—Dr. W. H. Galt retires from the editorial staff of this journal; his position is occupied by Dr. Lunsford P. Yandell, Jr.—*American Bi-Weekly.*

